# Socioeconomic inequalities in health problems in the first two years of life: Pelotas (Brazil) birth cohort, 2015

**DOI:** 10.1590/0102-311XEN208022

**Published:** 2024-10-18

**Authors:** Bruno Pereira Nunes, Thaynã Ramos Flores, Vanessa Iribarrem Avena Miranda, Bárbara Heather Lutz, Marília Cruz Guttier, Marysabel Silveira, Andréa Dâmaso Bertoldi

**Affiliations:** 1 Programa de Pós-graduação em Enfermagem, Universidade Federal de Pelotas, Pelotas, Brasil.; 2 Programa de Pós-graduação em Epidemiologia, Universidade Federal de Pelotas, Pelotas, Brasil.; 3 Departamento de Medicina Social, Universidade Federal de Pelotas, Pelotas, Brasil.

**Keywords:** Socioeconomic Factors, Healthcare Disparities, Multiple Chronic Conditions, Adverse Childhood Experiences, Birth Cohort, Fatores Socioeconômicos, Disparidades em Assistência à Saúde, Múltiplas Afecções Crônicas, Experiências Adversas na Infância, Coorte de Nascimentos, Factores Socioeconómicos, Disparidades en Atención de Salud, Afecciones Crónicas Múltiplas, Experiencias Adversas de la Infancia, Cohorte de Nacimiento

## Abstract

Brazil is characterized by an unfinished agenda of health inequalities, which impact health problems in the childhood. This study aimed to evaluate the socioeconomic inequalities of health problems in the early childhood. This is a prospective study, using data from the birth cohort carried out in the city of Pelotas (Rio Grande do Sul State, Brazil) in 2015. The outcomes were health problems presented at 12 and 24 months: cough, breathing difficulty, diarrhea, ear pain, pneumonia, urinary infection, hospitalization, and other health problems. Socioeconomic inequalities were measured applying the slope index of inequality (SII) and the concentration index (CIX), with wealth index and maternal schooling being the socioeconomic variables. The inequalities in the number of health problems were evaluated by Poisson regression. The perinatal sample comprised 4,275 children. At 12 months approximately 74% of the children presented 1 or more health problems, while at 24 months, approximately 44% presented 2 or more health problems. For all period, the mean number of health problems was 2.9 (standard deviation = 2.0). Higher frequencies were observed for children belonging to the poorest income quintile and with lower maternal education, except for 1 or more health problems at 24 months. The greatest absolute and relative inequality was observed for 2 or more health problems at 12 months (SII: -0.23, 95%CI: -0.29; -0.18 and CIX: -0.19, 95%CI: -0.25; -0.14). There is an opposite dose-response relation for the risk of accumulation of health problems according to maternal schooling (1.07, 95%CI: 1.04; 1.09) and wealth categories (1.03, 95%CI: 1.01; 1.04), in the full adjusted models. The study confirms inequalities due to health problems in Brazilian children, especially in the first year of life.

## Resumo

O Brasil é marcado por uma agenda inacabada em relação às desigualdades na saúde, impactando os problemas de saúde da infância. O objetivo deste estudo foi avaliar as desigualdades socioeconômicas relacionadas aos problemas de saúde da primeira infância. Este é um estudo prospectivo com base na coorte de nascimentos realizado na cidade de Pelotas (Rio Grande do Sul, Brasil) em 2015. Os desfechos foram problemas de saúde apresentados aos 12 e 24 meses de idade: tosse, dificuldade para respirar, diarreia, dor de ouvido, pneumonia, infecção urinária, hospitalização e outros problemas de saúde. As desigualdades socioeconômicas foram aferidas usando o índice de desigualdade absoluta (SII, acrônimo em inglês) e o índice de concentração (CIX, acrônimo em inglês), considerando o índice de riqueza e escolaridade materna como variáveis socioeconômicas. A regressão de Poisson foi utilizada para avaliar as desigualdades no número de problemas de saúde. Um total de 4.275 crianças foram incluídas na análise. Aos 12 e 24 meses, aproximadamente 74% e 44% apresentaram 1 ou mais e 2 ou mais problemas de saúde, respectivamente. Para todo o período, o número médio de problemas de saúde foi de 2,9 (desvio padrão = 2.0). Maiores frequências foram observadas para crianças no quintil de renda mais baixa e com menor escolaridade materna, exceto para 1 ou mais problemas de saúde aos 24 meses. A maior desigualdade absoluta e relativa foi observada para 2 ou mais problemas de saúde aos 12 meses (SII: -0,23, IC95%: -0,29; -0,18 e CIX: -0,19, IC95%: -0,25; -0,14). Com base nos modelos ajustados, foi encontrada uma relação dose-resposta oposta para o acúmulo de problemas de saúde com base na escolaridade materna (1,07, IC95%: 1,04; 1,09) e nas categorias de riqueza (1,03, IC95%: 1,01; 1,04). Portanto, o estudo confirma as desigualdades relacionadas aos problemas de saúde em crianças brasileiras, especialmente no primeiro ano de vida.

## Resumen

Las desigualdades sanitarias en Brasil afectan a la salud infantil. El objetivo de este estudio fue evaluar las desigualdades socioeconómicas de los problemas sanitarios de la primera infancia. Este es un estudio prospectivo de una cohorte de nacimientos realizada en la ciudad de Pelotas (Rio Grande do Sul, Brasil) en 2015. Los desenlaces fueron problemas sanitarios de niños de 12 y 24 meses de edad como tos, dificultad para respirar, diarrea, dolor de oído, neumonía, infección del tracto urinario, hospitalización, entre otros problemas. Las desigualdades socioeconómicas se midieron con el índice de desigualdad absoluta (SII, por sus siglas en inglés) y el índice de concentración (CIX, por sus siglas en inglés). El índice de ingresos y nivel educativo de la madre fueron las variables socioeconómicas. Las desigualdades en el número de problemas sanitarios se evaluaron mediante la regresión de Poisson. La muestra perinatal fue conformada por 4.275 niños. A los 12 y 24 meses, aproximadamente el 74% y el 44% de los niños tenían uno o más y dos o más problemas de salud, respectivamente. Durante el período, el número promedio de problemas sanitarios fue de 2,9 (desviación estandar = 2.0). Se observaron frecuencias más altas para los niños pertenecientes al quintil de ingresos más pobres y con el nivel educativo de la madre más bajo, a excepción de uno o más problemas de salud a los 24 meses. La mayor desigualdad absoluta y relativa se observó en dos o más problemas sanitarios a los 12 meses (SII: -0,23, IC95%: -0,29; -0,18 e CIX: -0,19, IC95%: -0,25; -0,14). Con base en los modelos ajustados, se encontró una relación dosis-respuesta opuesta para la acumulación de problemas sanitarios en cuanto al nivel educativo de la madre (1,07, IC95%: 1,04; 1,09) y a los ingresos (1,03, IC95%: 1,01; 1,04). Este estudio confirma las desigualdades por problemas sanitarios de niños brasileños, especialmente en el primer año de vida.

## Introduction

Recent evidence has shown an association between childhood health, chronic diseases, and adult multimorbidity ^1^ ^,^ ^2^ ^,^ ^3^. Social, economic, cultural, ethnic/racial, psychological, and behavioral factors influence the occurrence of health problems ^4^. Social determinants play a decisive role, making it possible to observe the unequal health conditions of different groups, even in differentiated access to the health system ^5^ ^,^ ^6^ ^,^ ^7^ ^,^ ^8^. A study by Russell et al. ^9^ showed that cumulative socioeconomic disadvantage increases the potential for developing chronic illness in early childhood.

The most common health problems in early childhood are acute and infectious diseases, particularly in the respiratory and digestive systems. A recent systematic review has shown an association between asthma and later development of chronic obstructive pulmonary disease (COPD) (OR = 7.23, 95%CI: 5.05; 10.33) ^3^. A possible explanation (which has not been confirmed) is that airway inflammation and remodeling caused by uncontrolled asthma can progress from reversible bronchospasm to irreversible airway obstruction ^10^. The first thousand days of life are associated with symptoms of asthma in a Brazilian cohort conducted in São Luís, Maranhão State ^11^.

Stressful factors, such as divorce of parents, spending time in the hospital, among others, occurring in childhood and adolescence were associated with the occurrence of cardiovascular disease in adulthood (OR = 2.14; 95%CI: 1.56; 2.94), although the pathophysiological mechanisms have not been fully clarified ^12^. A study including 4,731 older adults found a strong association between the presence of stressful factors in childhood and multimorbidity ^1^.

Understanding events that interfere with children’s health and identifying individuals with the greatest burden of childhood morbidity can help to prioritize and carry out interventions to prevent later chronic diseases ^7^ ^,^ ^13^ ^,^ ^14^. The progressive process of population aging, increasing exposure to environmental factors, changes in lifestyles, and progress in the effectiveness of health care have led to an increase in the prevalence of noncommunicable diseases globally. Multimorbidity has become a priority for public health considering its prevalence, severity, impact on quality of life, and management ^15^. Furthermore, most studies evaluating health inequities are cross-sectional and conducted with adults or older adults ^15^. We consider that this study can contribute to this sense, by analyzing inequities using robust indices to understand the relation between health problems in childhood and socioeconomic factors.

Therefore, this study aimed to evaluate the socioeconomic inequalities of health problems in the first two years of life (up to 24 months of age) in the 2015 Pelotas (Rio Grande do Sul State, Brazil) birth cohort.

## Methods

### Sample and study design

This is a prospective cohort study, containing data from the 2015 birth cohort, which included all births that occurred in the city of Pelotas from January 1 to December 31, 2015. Mothers living in the urban area of the municipality were included. Individuals born in this period whose mothers resided in the district of Jardim América and Colônia Z3 were also included, maintaining comparability with the other birth cohorts carried out in Pelotas (1982, 1993, and 2004).

### Data collection and instruments

Participants’ mothers were interviewed shortly after childbirth in the hospital, and were invited to join the perinatal study, addressing various aspects of prenatal care, socioeconomic and demographic profile, in addition to maternal, household, and newborn characteristics ^16^. Following the 2015 cohort, other studies took place at three, 12, 24, and 48 months. At three and 12 months, the interviews were carried out in the households, while most of the interviews at 24 and 48 months were carried out at the Health Research Center clinic. A team of interviewers was properly trained and qualified for the work. In addition to questions, measurements were taken at all follow-ups, including weight and height of both the mother and the child ^15^. This study used variables from the follow-ups during perinatal, and at three, 12, and 24 months (Supplementary Material, Figure S1: https://cadernos.ensp.fiocruz.br/static//arquivo/suppl-e00208022_5265.pdf).

### Ethical aspects

The 2015 birth cohort project was submitted to and approved by the Research Ethics Committee of the School of Physical Education and Physical Therapy of the Federal University of Pelotas (UFPel, protocol n. 26746414.5.0000.5313). All mothers interviewed signed an informed consent form, agreeing to participate in the study.

### Outcomes

Outcomes were health problems presented by the children at 12 and 24 months of age, reported by the mother or guardian during interviews. The outcomes were operationalized by the following questions, in both follow-ups: cough “Has <CHILD> had any cough since <day of the week> last week?”; breathing difficulty “Has <CHILD> had difficulty in breathing since <day of the week> last week?”; diarrhea “Has <CHILD> had diarrhea since <day of the week> two weeks ago?”; Any other health problem “Has <CHILD> had any other health problem since <day of the week> two weeks ago?”; ear pain “Has <CHILD> ever had ear pain?”; pneumonia “Has <CHILD> ever had pneumonia?”; urinary infection “Has <CHILD> had any urinary infection since birth?”; hospitalization “Has <CHILD> been admitted to hospital since birth?” (considered a marker of health problems). At the 24 months follow-up the questions for ear pain, pneumonia, urinary tract infection, and hospitalization refer to the last follow-up (if after 12 months the child had presented any of these problems). The 2015 cohort questionnaires are available at: http://www.epidemio-ufpel.org.br/site/content/coorte_2015/questionarios.php.

For analysis purposes, outcomes were grouped into ≥ 1 (at 12 and 24 months), ≥ 2 (at 12 and 24 months), and number of health problems (up to 24 months).

### Main exposures

The main exposures of this study were maternal schooling in complete years (0-4; 5-8; 9-11; 12 or more) and wealth index based on family income in Brazilian currency (in quintiles: first/poorest quintile and fifth/richest quintile), both collected in the perinatal wave.

### Covariates

Some independent variables collected in the perinatal study were included in the model as confounders: gestational age at birth (in days), child sex (female/male), and birth weight (in grams). Also, a variable collected in the follow-up of three months of age was included: breastfeeding status (exclusive/predominant/partial/weaned).

### Statistical analysis

Descriptive analyses were performed using Stata 15.0 (https://www.stata.com) to obtain the prevalence for categorical variables and means with respective standard deviations (SD) for numerical variables.

Two indexes were calculated in order to measure inequalities: the slope index of inequality (SII) and the concentration index (CIX). The SII shows the absolute difference, in percentage points, between extreme coverage, in this case, mothers with less (0-4 years) and more (12 years or more) years of education, as well as the poorest and richest quintile, using a logistic regression model. The CIX is based on a scale ranging from -1 to +1, in which zero represents an uneven distribution, both in education and family income. Positive CIX values suggest that the distribution is in favor of more educated and wealthier mothers. The SII presents absolute inequality while the CIX measures relative inequality ^17^.

The inequalities of the number of health problems were evaluated by Poisson regression, obtaining the prevalence ratio (PR) and respective 95% confidence intervals (95%CI). The adjusted analysis considers two models to evaluate the dose-response relation between the number of health problems (up to 24 months of age) and the maternal education and family income. In Model 1, the analysis was adjusted for sex and socioeconomic indicator (maternal schooling or wealth). In Model 2, the adjusted analysis includes variables from Model 1 plus breastfeeding, gestational age, and birth weight. These variables were included in the model to obtain adjusted PR for the main association based on the literature ^7^ - there are no confounders selection by statistical assessment. Prediction calculation was also performed using the margins and marginsplot commands to predict the adjusted number of health problems up to 24 months of age, according to maternal education and family income. Associations with p-value < 0.05 were considered statistically significant.

## Results

The initial sample consisted of 4,275 children, with the smallest sample size analyzed being 3,857 children (adjusted models − outcome number of health problems up to 24 months). The original and analyzed sample were similar. The mean maternal age was 27.6 years (SD = 0.10). Half of the sample was female, with an average of 269 days of gestational age and 3,171 grams of birth weight. The mean income was close to BRL 3,000.00 (SD = 4,361). Most mothers had 9 years of schooling or more (65.2%). At 3 months, 44.7% of the children were exclusively breastfed. At 12 and 24 months, 74.1% and 73.9% had some of the health problems assessed, with cough and ear pain being the most frequent. The frequency of two or more health problems was similar at 12 and 24 months, approximately 44%. At 24 months, children had a mean of 2.9 health problems (SD = 2.0) (Table 1).

**Table 1:** Sample description. 2015 Pelotas (Brazil) birth cohort.

Characteristic	%
Perinatal (n = 4,275)	
Maternal age (years) [mean (SD)]	27.6 (6.6)
Female sex	49.4
Gestational age (days) [mean (SD)]	269 (16)
Birth weight (g) (n = 4,259) [mean (SD)]	3,169 (564)
Wealth, family income (BRL) (n = 4,273) [mean (SD)]	3,064 (4361)
Maternal schooling at birth (years) (n = 4,274)	
0-4	9.2
5-8	25.6
9-11	34.1
12 or more	31.1
Three months	
Breastfeeding (n = 4,102)	
Exclusive	44.7
Predominant	7.4
Partial	24.4
Weaned	23.5
12 months	
Health problems (n = 4,017)	
Cough	39.1
Breathing difficulty	22.5
Diarrhea	13.6
Other health problem	15.1
Ear pain	32.6
Pneumonia	7.5
Urinary infection	3.4
Hospitalization (n = 4,011)	16.8
1 or more problems	74.1
2 or more problems	44.2
24 months	
Health problems (n = 4,011)	
Cough	43.3
Breathing difficulty	22.2
Diarrhea	14.8
Other health problem	15.3
Ear pain	30.6
Pneumonia	5.9
Urinary infection	4.1
Hospitalization (n = 4,002)	7.9
1 or more problems	73.9
2 or more problems	43.5
Number of health problems * (12 + 24 months) (n = 3,892 **)	2.9 (2.0)

* Sum of all health problems reported up to 24 months;

** Valid information to health problems up to 24 months.

**Figure 1:**
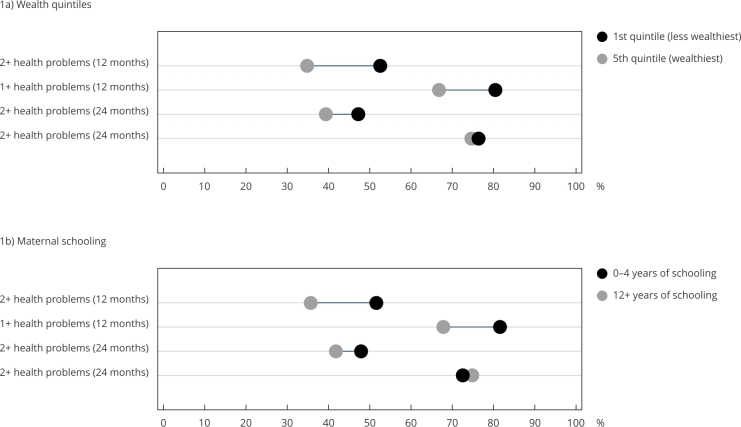
Health problems at 12 and 24 months according to extremes of wealth quintiles and maternal schooling. 2015 Pelotas (Brazil) birth cohort.

At 12 months, concomitant health problems were higher among children belonging to the poorest income quintile (p < 0.001) and with lower maternal education (p < 0.001). At 24 months, the occurrence was higher among children with lower purchasing power (p = 0.025) and maternal education (p = 0.015) for 2 or more health problems. There was no statistically significant difference for the occurrence of 1 or more problems according to income and maternal education at 24 months (Figure 1).

We observed an absolute and relative inequality for problems at 12 months and for two or more problems at 24 months. The highest magnitude was observed for two or more problems at 12 months, for absolute inequality according to maternal education (SII: -0.23) and family income (SII: -0.19) (Table 2).

**Table 2:** Absolute (slope index of inequality − SII) and relative (concentration index − CIX) inequalities according to maternal schooling and wealth. 2015 Pelotas (Brazil) birth cohort.

Health problem	Maternal schooling		Wealth, family income	
	SII (95%CI)	CIX (95%CI)	SII (95%CI)	CIX (95%CI)
12 months				
1 or more	-0.19 (-0.24; -0.14)	-0.04 (-0.05; -0.03)	-0.18 (-0.22; -0.13)	-0.03 (-0.05; -0.02)
2 or more	-0.23 (-0.29; -0.18)	-0.08 (-0.10; -0.06)	-0.19 (-0.25; -0.14)	-0.06 (-0.08; -0.04)
24 months				
1 or more	0.00 (-0.05; 0.05)	0.00 (-0.01; 0.01)	-0.02 (-0.07; 0.03)	-0.01 (-0.02; 0.01)
2 or more	-0.08 (-0.13; -0.02)	-0.03 (-0.05; -0.01)	-0.09 (-0.14; -0.03)	-0.03 (-0.05; -0.01)

*Note: 95%CI: 95% confidence interval.

When adjusting the analyses for sex and socioeconomic indicators, the associations were maintained only for 2 or more problems at 12 months, even after additional adjustment for breastfeeding, gestational age, and birth weight. The number of health problems up to 24 months showed a linear association with education and income regardless of the adjustment (Table 3).

**Table 3:** Crude and adjusted analysis of health problems indicators and socioeconomic variables. 2015 Pelotas (Brazil) birth cohort.

Health problem	Maternal schooling *			Wealth, family income *		
	Crude	Adjusted 1	Adjusted 2	Crude	Adjusted 1	Adjusted 2
	PR (95%CI)	PR (95%CI)	PR (95%CI)	PR (95%CI)	PR (95%CI)	PR (95%CI)
12 months						
1 or more	1.07 (1.03; 1.12)	1.05 (1.00; 1.10)	1.04 (1.00; 1.09)	1.05 (1.02; 1.08)	1.03 (1.00; 1.06)	1.03 (1.00; 1.06)
2 or more	1.16 (1.11; 1.22)	1.11 (1.05; 1.18)	1.11 (1.05; 1.18)	1.10 (1.06; 1.13)	1.04 (1.01; 1.09)	1.04 (1.01; 1.09)
24 months						
1 or more	1.00 (0.96; 1.04)	0.99 (0.95; 1.03)	0.99 (0.95; 1.03)	1.01 (0.98; 1.03)	1.01 (0.98; 1.04)	1.01 (0.98; 1.04)
2 or more	1.05 (1.00; 1.11)	1.03 (0.97; 1.09)	1.03 (0.97; 1.10)	1.04 (1.01; 1.08)	1.03 (0.99; 1.07)	1.03 (0.99; 1.07)
Number of health problems (12 + 24 months)	1.09 (1.07; 1.11)	1.06 (1.04; 1.09)	1.07 (1.04; 1.09)	1.05 (1.04; 1.07)	1.03 (1.01; 1.05)	1.03 (1.01; 1.04)

95%CI: 95% confidence interval; PR: prevalence ratio.

Adjusted 1: sex and socioeconomic indicator (maternal schooling or wealth); Adjusted 2: adjusted 1, breastfeeding, gestational age, and birth weight.

* Dose response relation. prevalence ratio (PR) represents the linear risk according to the decrease of the maternal schooling and wealth categories.

**Figure 2:**
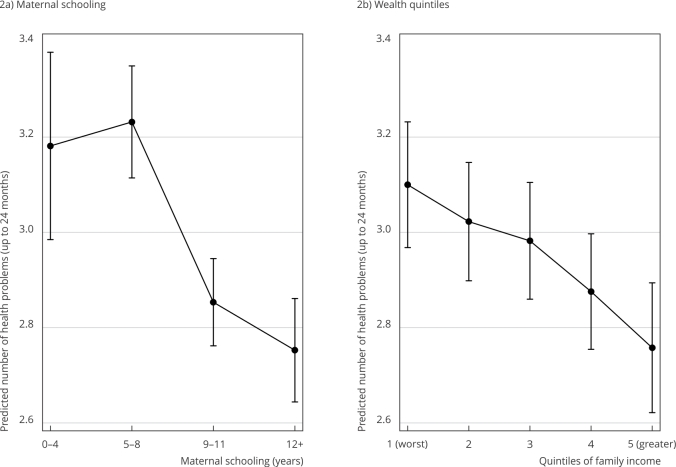
Predicted number of health problems up to 24 months of age according to maternal schooling and wealth (quintiles of family income). 2015 Pelotas (Brazil) birth cohort.

After adjustment, children with less and more educated mothers had on average 3.2 and 2.7 health problems, respectively. Children with lower purchasing power presented 3.1 health problems versus 2.8 for those in the richest quintile (Figure 2). Sensitivity analysis was performed including mode of delivery and the results were virtually equal.

## Discussion

This evidence suggests inequalities in the occurrence of health problems, mainly related to the accumulation of morbidities in the first year of life. We observed absolute and relative inequalities for both ages (12 and 24 months). In addition, the average number of health problems was higher among children whose mothers had less years of education and who belonged to lower-income families, with a dose-response relation.

The observed inequalities confirm the body of evidence on the influence of social determinants on the health situation of populations, and the mechanisms may include intergenerational transmission of poverty, syndemic, intrauterine exposures, and epigenetics. Identifying a greater occurrence of health problems among children belonging to families with lower purchasing power is in line with studies on the long-term effect of socioeconomic inequalities in the higher occurrence of chronic diseases in adulthood ^18^ ^,^ ^19^ ^,^ ^20^. In addition to other factors throughout the life cycle, such as healthy habits and obesity, the greater burden of acute problems in childhood may be associated with the chance of a chronic low-grade inflammation in adulthood, indicating another possible causal path for the cumulative effect of socioeconomic position on the pathophysiology of chronic diseases and mortality risk ^3^ ^,^ ^21^ ^,^ ^22^.

The observed inequalities are related to the living conditions of families who experience difficulties providing adequate care and protection for health problems ^23^, mainly respiratory ones. Pelotas is a city with high levels of humidity and severe winter, which makes quality housing, public transportation, public education, and access to goods and services even more necessary, ensuring, as much as possible, the protection of children from respiratory problems. On the other hand, a factor that may attenuate these inequalities is the protection generated by exclusive breastfeeding, which is greater among children with lower purchasing power (55% vs. 28%, and 57% vs. 34%, comparing the extremes of maternal education and income quintiles, respectively). Even so, this possible protection does not seem to be sufficient to mitigate the differences between children from different socioeconomic positions.

In addition, children up to 12 months of age may have a lower immune response when compared to those aged 24 months, that is, they may be more susceptible to health problems. In this study, lower maternal education and lower income were statistically associated with two or more health problems among children aged 12 months. Not only a lower immune response but also socio-economic conditions may influence child health. Studies have identified that children from families with lower socio-economic status, less education, or even mixed-race or black skin color presented a higher risk of unfavorable health conditions ^24^ ^,^ ^25^. The remarkable difference found in the first year of life signals the impact of socioeconomics on health status before health interventions which seem to reduce inequalities over time (e.g., vaccination and respiratory problems up to 12 months). Despite the advances of the Brazilian health system in improving maternal and child health conditions ^26^ ^,^ ^27^, actions to reduce poverty and social inequalities (the cause of all causes) ^28^ are still necessary to avoid medium- and long-term effects of increased exposure to health problems in early life.

Hospitalization and pneumonia were health conditions whose prevalence drew our attention, especially among 12-month-old children. However, other problems such as coughing and breathing difficulty, which are part of the diagnosis for asthma, were also alarming for both 12-month and 24-month-old children. Regarding hospitalization, a study carried out in the 2004 Pelotas cohort observed similar prevalence to those identified in this study. This prevalence was reduced at four and six years of age ^7^. Pneumonia is a major cause of hospitalization among children ^7^ ^,^ ^29^ ^,^ ^30^, and in some more severe cases, it can progress to death, especially among children under 12 months of age ^29^ ^,^ ^31^. However, after vaccination against the main causative agents of the disease (12 months), the prevalence of pneumonia decreased considerably, remaining stable or without a notable reduction among children from families with lower purchasing power ^32^.

It should be noted that our study may hold some limitations. The possibility of a recall bias due to the temporality of the questions should be considered, which could underestimate our findings. However, we believe that all health problems investigated are very marked at this stage of life (children up to two years old). Another limitation could be the use of cough and breathing difficulty in the score for health problems together with pneumonia and hospitalization, considering that these two problems are part of the diagnosis of asthma. Nevertheless, the association was maintained when we performed sensitivity analyses, removing cough and breathing difficulty from the health problem score. Finally, all health problems were reported by the mother or guardian, and we did not perform objective measures (medical records, for example) to assess these health problems among the children.

Some positive points of this study should be highlighted. The first is being a longitudinal study that enables the monitoring the development of early childhood diseases. The data obtained can contribute to the prevention of chronic diseases, as well as to the cautious evaluation of respiratory diseases throughout the life cycle. In addition, no differences were observed from the original sample to the analytical one, which assures the study regarding the selection bias.

## Conclusion

This study confirms inequalities in the occurrence of health problems among Brazilian children. Macro- and micro-level public policies are needed to decrease the occurrence of diseases and its inequalities over life-course, including actions in the health systems and services to prevent avoidable diseases, in addition to intersectoral policies to guarantee the children’s safety development. To sum up, this study highlights the relevance of the concomitant assessment of problems that better capture the socioeconomic differences in health problems in children up to 24 months of age.
